# From client to co-worker: a case study of the transition to peer work within a multi-disciplinary hepatitis c treatment team in Toronto, Canada

**DOI:** 10.1186/s12954-018-0245-7

**Published:** 2018-08-14

**Authors:** Paula Tookey, Kate Mason, Jennifer Broad, Marty Behm, Lise Bondy, Jeff Powis

**Affiliations:** 1 South Riverdale Community Health Centre, 955 Queen St East, Toronto, ON M4M 3P3 Canada; 2 Department of Medicine, Division of Infectious Diseases, Schulich School of Medicine and Dentistry, Western University. St. Joseph’s Health Care, 268 Grosvenor Street, London, ON N6A 4V2 Canada; 3 Michael Garron Hospital, 835 Coxwell Ave, Toronto, ON M4C 3E7 Canada

**Keywords:** Peer work, Hepatitis C, Multi-disciplinary teams, Case study

## Abstract

**Background:**

Despite the integration of peer workers into harm reduction services, there is little documentation regarding the experience of this integration or of models in which peers are fully integrated as members of health care teams. The purpose of this study was to gain an in-depth understanding of the transition from client to support worker from the perspective of two individuals who received treatment for hepatitis C at a multi-disciplinary, community-based program, grounded in a harm reduction approach to substance use.

**Methods:**

A participatory case study design was selected. Interviews were conducted with two current peer workers who were also involved in the study design, analysis and writing. Data was coded and analyzed using an inductive approach to identify emergent themes.

**Results:**

Five primary themes emerged during our analysis of the facilitators and challenges of the transition from client to support worker: (1) the role of prior experience, (2) changes in substance use practices, (3) shifts in relationships with community members and friends, (4) supportive organizational and structural factors, and (5) role transition as a journey. In some cases, themes overlapped and contained elements that were both facilitating and challenging.

**Conclusions:**

The transition from client to co-worker is a gradual process and one that is supported by, and in turn helps to support, a number of other personal transitions. The cases examined here suggest that a model of peer employment with broad qualification criteria, sufficient transition timelines, flexible job responsibilities, a solid investment in the inclusion of people with lived experience, and a harm reduction framework will support successful integration of current and/or former clients into health care teams.

## Background

### Peer work and hepatitis C

The involvement of people who use drugs in the development and provision of harm reduction services is increasingly common and widely recognized as best practice [1]. People who use drugs have historically been central to the initiation of harm reduction efforts [2] from syringe and other drug use equipment distribution, to supervised injection, take-home naloxone programs, disease prevention workshops, community outreach, and drug policy advocacy groups [3–9]. Despite how integral people who use drugs have been to the establishment and ongoing success of harm reduction efforts, their formal and full involvement within such programs and services is often limited, particularly in more medicalized settings. The integration and involvement of people who use drugs as workers in drug treatment programs, in health care organizations, or within programs that treat viral infections associated with injection drug use such as hepatitis C (HCV), are rare [6, 10, 11]. A recent review of peer support models for HCV found that few studies have been published describing practical experience with peer models in the management of the HCV infection outside of opiate substitution therapy settings [12].

HCV is one of the most common viral illnesses worldwide, with recent estimates suggesting over 71 million people are living with the infection [13]. It has been estimated globally that 67% of people who inject drugs are HCV antibody positive [14], yet only 1–8% of HCV positive substance users receive treatment [15–18]. Barriers to care are many and remain prevalent despite the recent advent of treatments with improved efficacy and tolerability. At a systems level, tertiary care centers where HCV care is predominantly delivered are often not well-equipped to provide the flexibility and support required to address complex needs related to poverty, social marginalization, and mental health comorbidities [19, 20]. Poor health literacy, past negative experiences with the health care system, and competing health or social priorities also prevent some individuals from accessing care [19–24]. Stigma associated with HCV due to its association with injecting drug use, and discrimination in the health sector towards people who use drugs are also important barriers to accessing treatment [12, 25, 26].

Low-barrier, multi-disciplinary, harm reduction based models of HCV treatment, which emerged in the last decade in response to the gaps in treatment access, have demonstrated success in engaging people who use drugs in HCV care with outcomes that are comparable to clinical trials [27–29]. Many of these community-based models feature some form of peer involvement, primarily as educators, but also in system navigation or outreach roles [12, 30, 31]. Increasingly, the benefits of peer involvement in HCV care have been recognized, and peer involvement is now a recommended component of HCV prevention and treatment guidance documents [32, 33]. Some of the documented benefits of peer involvement in HCV prevention and care include improved client attendance and compliance with medical recommendations [34], improved retention of particular sub-groups in HCV treatment [35], improved relationships between clients and healthcare providers [36] and service delivery transformation [12]. In most cases, peer workers provide one-on-one support within opioid substitution treatment (OST) clinics or other drug treatment settings [12, 37]. Despite the improved integration of peers into HCV care delivery, there is little documentation regarding models in which peers are fully integrated as members of a primary health care team or the perceptions of peer workers themselves [38]. Furthermore, little research has been conducted with people with lived experience of HCV as co-investigators. The purpose of this participatory case study was to gain an in-depth understanding of the facilitators and challenges in the transition from client to support worker from the perspective of individuals who underwent this transition within a multi-disciplinary, primary-care based HCV treatment program.

### Program model

The Toronto Community Hep C Program (TCHCP) began in 2007 in response to many of the gaps and barriers to health care for marginalized people living with HCV noted above. The program is a partnership between three community-based health centers with integrated on-site specialist support from a nearby hospital. HCV treatment is delivered alongside a weekly education and support group held at each of the three partner sites. Primary care clinicians (physicians, nurses, and nurse practitioners) are available during the weekly group sessions with monthly on-site support from the HCV treatment specialist. Clients receive both HCV-specific and more general primary care. They may also access case management, counseling, or other one-on-one supports and referrals through the group facilitators or other program staff. In addition to the treatment group, the program also offers a drop-in group for clients who have completed treatment or who are not eligible for or currently able to do treatment, as a way of maintaining their engagement in care. The program has demonstrated positive treatment outcomes that are comparable to clinical trials in hospital-based settings [27, 28].

The TCHCP is based on the theories and practices of harm reduction and community development, both of which value the involvement of people with lived experience in decision making and service delivery. These program model theories have been previously described [39]. The TCHCP operates from the position that involving people with lived experience of hepatitis C in its program development is essential to both program quality and positive health outcomes and has worked towards the meaningful inclusion of people with lived experience since its inception. These principles and practices have also been drawn from and owe much to the “Nothing About Us Without Us” and GIPA/MEPA (greater involvement and meaningful engagement of people living with HIV/AIDS) political and activist movements [40, 41]. Peer-to-peer support and involvement are currently integrated into the program in several ways: group support, a patient advisory board, and community support worker positions filled by current or former program clients.

### Group support

Each type of TCHCP group offered by the program is established as a welcoming space where a sense of community and mutual support are nurtured. The group is an important place of belonging and peer-to-peer support and knowledge sharing. Clients who are further along in their treatment process act as resources and mentors for newer members. Those who have recently finished treatment are able to offer encouragement and reassurance to individuals who are experiencing side-effects or other life challenges while on treatment. Through the program’s groups, participants learn about and get connected to other community resources*.* The program encourages collective action and organizing and aims to equip clients with the necessary skills for self-advocacy, as well as critical awareness at a personal, program, and community level.

### Patient advisory board

The program’s Patient Advisory Board (PAB) was established in the fall of 2010 to enable the meaningful participation of people living with HCV in the development, implementation, and evaluation of the program. PAB members participate in partnership meetings, presentations, workshops, training, and strategic planning. The PAB is made up of two group members from each site who are elected by other group members and aims to be representative of the diversity of program clients. The PAB meets monthly or more, when needed, and members are paid an honorarium for their time at meetings and other events. The PAB has three main roles: (1) to provide feedback on existing program components, practices, and issues; (2) to provide guidance and input into program development, research, evaluation, and training; (3) to conduct public education and awareness regarding HCV through presentations and other public speaking engagements.

### Community support workers

Since August 2011, the program has annually offered a Community Support Worker (CSW) training program for up to 12 current or former TCHCP clients. The purpose of this training is to further increase the capacity of program clients to act as support workers for their peers within the program or at other agencies elsewhere in the community. The training program consists of approximately 16 weekly, 2-hour sessions focused on skills-building and topics such as communication, self-care, boundaries, and advocacy. The HCV Case Manager (study author, Paula Tookey) provides overall development and coordination for the training program including facilitating several of the training sessions and providing one-on-one support for participants. The training program was advertised by flyers that were circulated at the TCHCP groups and posted throughout the health centers. Past clients who had previously expressed interest in employment opportunities were contacted by phone and told about the training. Interested clients applied in writing by submitting a brief and informal (could be hand written) letter of interest to the training coordinator. The number of training spots was capped at 12 based on the coordinator’s past experience of running a variety of psycho-educational and training programs; to allow for adequate group discussion and cohesion and to ensure sufficient staff capacity to identify and support individual learning and training needs. To date, the program has received approximately 12 applications per training cycle and as such has been able to accommodate all clients who applied.

In June 2012, six peer training program graduates were hired for two HCV CSW positions at each of the three program sites. The goal of these positions is multi-fold: to provide a meaningful employment/skill building opportunity for clients, to keep the program grounded in and responsive to the needs of the community, and to fill a program need for additional client support/education. The work of these positions varies from worker to worker and has evolved for each individual over time. Responsibilities and tasks remain flexible to allow for varying stages of job readiness and worker interest. CSW responsibilities currently include group facilitation, public speaking, training of other peer workers, client accompaniment to appointments, administrative program support, and informal one-on-one client support. CSWs are employees of South Riverdale Community Health Centre which is not a unionized organization and are paid an hourly wage, vacation pay and sick time. They work a minimum of 6 hour per week. As much as possible (limited primarily by hours of work), CSWs are fully integrated members of the multi-disciplinary HCV program/team: attending meetings, participating in case conferencing, independently representing the program externally, attending conferences and professional development workshops, and contributing to program planning and research. CSWs receive practical support, supervision and mentorship from the HCV case manager who works closely with the CSWs on most activities. Support is integrated during service delivery or program activities, as well as in pre- and post-shift discussions. While client care provision through the TCHCP is time limited (clients leave the program 12-weeks post end of treatment), CSWs may continue to be primary care and/or harm reduction clients at one of the three health centers where the TCHCP is offered. Wherever possible, the program attempts to separate where CSWs work from where they receive care. With three locations, the program has some ability to ensure CSWs do not work (or work very little) in the health center where they continue to receive health care or will help them to find primary care elsewhere if desired. Authors Jennifer Broad and Marty Behm have both been in the CSW role since it began and are the only workers from the original group of six who were still in the role. Two from this original group had gone on to full-time employment in other sectors. Pre-existing alcohol use or mental health issues became problematic for the two remaining workers who had to leave the role to prioritize this aspect of their health.

## Methods

A participatory approach was chosen for this study. The study idea was generated by the program’s patient advisory board and CSWs who were interested in using research as a tool for exploring and detailing the experiences of peer workers. Everyone on the study team, including the CSWs who ultimately became participants of the study, as well as co-researchers, had an equitable contribution and were involved in all stages of the design and analysis [42]. This approach aligned with the TCHCP’s collaborative structure and view of service delivery, which has been described above. The study was approved by the Research Ethics Board at Michael Garron Hospital.

### Design

A case study design was selected as the best method for undertaking a more holistic investigation into how and why some individuals successfully make the transition from client to peer worker. It was the study team’s hypothesis that factors would be complex, and the selected methodology allowed for in-depth exploration, as well as diverse and contextual perspectives [43]. The case study design was also selected for its accessibility for study team members without formal research training and as a methodology that lends itself to practical application [44]. A case study approach allowed for and encouraged subjective perspectives, as well as deep contextual understanding, expertise that each member of the research team could offer equally. The ability to include and consider both the uniqueness and complexity of each person’s experience was an important part of the design choice. This methodology allowed the research team the opportunity to explore the how and why, which was also important for a group of mainly frontline workers/clinicians who are primarily concerned with clinical practice implications.

### Sample

Two participants were purposefully drawn from the group of five community support workers who were employed by the TCHCP at the time of data collection. Authors Jennifer Broad and Marty Behm self-selected for the project out of an interest both in taking a more active research role within the program and in examining their own personal experiences. Jennifer was still a TCHCP client (for her second round of treatment) when she began her employment as a CSW and both continue to be primary care clients at one of the health centers where the TCHCP takes place. Jennifer and Marty had been patient advisory board members and were two of the original six peer workers hired with the program. As such, it was felt that they had sufficient program exposure and work experience to adequately comment on the study questions. The study team (including Jennifer and Marty) also felt that they best represented the program’s client diversity in terms of personal characteristics, background, and trajectories. Multiple conversations took place with Marty, Jennifer, and the rest of the study team prior to initiation and throughout the analysis and writing regarding the potential ramifications of their open participation. Both Jennifer and Marty already had considerable experience as public speakers where they have shared similar life details. They also felt strongly that any negative personal impact would be outweighed by the potential community benefit of reducing stigma and contributing to an understanding that people who use drugs are equal and valuable contributors to their communities. Jennifer and Marty viewed their participation in this paper as a form of activism, and felt that being open and vulnerable is the right thing to do even at some possible risk to them. Both also feel protected somewhat by their age and certain knowledge of their future goals, which do not include working in places where their current or past substance use would be judged negatively. The study team also undertook a lengthy process of reflection on the transcripts and revision of the manuscript. Decisions on what to include were ultimately made by Jennifer and/or Marty.

### Data collection

A formal informed consent process took place prior to the interviews and any audio recording. Written consent was obtained, and interviews were conducted by a health care provider (Lise Bondy) not otherwise affiliated with the program in the fall of 2015. This decision was made to allow for peer workers to be critical about the transition in ways that they might not have felt comfortable to do with their colleagues. The involvement of an external co-investigator also provided an objective perspective that helped validate and foster the reflexivity of the rest of the research team. Although participants were also co-researchers, the consent process was structured as it is for other potential research participants to ensure that the same rights and concerns were addressed. As part of the consent process, the participants/co-researchers were reminded that they should be careful with the information that they shared and assured that they would be able to remove any sensitivities from transcripts and/or this manuscript. Consent to be audio recorded was optional and was a separate part of the consent form. Participant/co-researchers understood that the study findings would be reported internally and would be submitted for publication in a peer-reviewed journal. Participants/co-researchers were also informed that they could withdraw from the study at any time and that nothing would be included in any reports or presentations without their permission. Interviews were guided by a set of semi-structured, open-ended questions and took approximately 55 min each. Questions covered each participant’s personal background, TCHCP client experience, and CSW experience. The interview questions and schedule were collectively designed and agreed upon by the study team.

### Analysis

Audio recordings of the interviews were transcribed verbatim. Only investigators participating in the analysis had access to the transcripts and recordings. Data collected from the interviews was coded and analyzed using an inductive approach to identify emergent themes. We employed a cross-case analysis to identify common and unique themes. A collective process of organizing and sorting the data was undertaken. One of the authors with experience in qualitative analysis provided a training session and guidance throughout the process. Authors PT, KM, JB, and MB initially reviewed the transcripts on their own for first impressions and then re-read for patterns. At this point, the authors began a series of group discussions interspersed with individual re-readings of the transcripts to compare notes, revise, and finally refine the emerging themes and concepts. These authors met six times over a period of 6 months to compare, review, and discuss findings. The focus of analysis was on uncovering both unique and common features of each transition from client to coworker and on the factors that were challenges or facilitators in this transition. From these discussions, the study team extrapolated and identified those factors that seemed to support the successful transition in these cases. The case presentations were initially pieced together from the formal interviews and were revised in subsequent discussions during data analysis. Sections were included to give the reader a sense of each person’s background and to provide relevant context to help frame and support our qualitative analysis. The HCV treatment experience itself was a significant life turning point for both participants and as such is a large part of each story. Each participant re-worked and finalized their own story. Writing of the manuscript was coordinated by a single author, but writing was often collaborative with sections revised and edited in group meetings. This process was time consuming, but ultimately produced what the study authors hope is a manuscript that is useful and accessible to academics and non-academics alike.

## Case presentations

### Marty’s story

I am 48 years old and was diagnosed with hep C in 2003 or 2004. I fought so hard to be able to do hep C treatment. I had to travel all across Canada. I was denied treatment about four times because I was an active drug user and they all wanted me to be abstinent for like six months. I tried different treatment programs but never lasted. It took four years until I finally made it to Toronto and to a nurse who led me to the [Toronto Community Hep C] Program. I had been shot down so much by then I was basically scared of health care. I did not want go in and get rejected again just because I smoke a joint or do a smash or drink a beer. Once I found the program it was still about two and a half years before I could start treatment because they found out that I also have HIV. So I had to build myself up, my immune system, because it was the old system of interferon back then, so that made it a lot rougher. The treatment really sucked – I had a lot of puking, I lost hair, I was nauseous a lot. You were emotionally and physically all over the place on that stuff [interferon]. I could not give up on it though. I’d given up on too many things. Giving up on treatment would have meant giving up on myself.

When I came to the program I was homeless, transient, all over the place. I was using drugs every day, all day. The program helped me find respite housing for a few months while I was on treatment and that really helped – so did the group, the camaraderie … and the meal! I got involved in the first Patient Advisory Board to help other people who were co-infected to access treatment, to stand up for other people in the community and to try to give everyone a voice in how they are treated – especially people who are marginalized. I was unsure at first about the peer training. I did not think I would ever get a job or even accepted into the training. But I wanted to try — to give back to the community and to myself.

Right now one of the biggest parts of my job is running the weekly Continuing Care Group, for clients who have gone through the treatment or who are not eligible for treatment right now. It’s a place for people to come and socialize and get support – so they can stay engaged with health care and harm reduction education. For many people it’s the highlight of their day. I like giving back to the community – not being looked at as a burden. I love the challenge of this job. It’s constantly changing, the people are constantly changing. I do not get bored. Having the community look up to me … I have improved my own lifestyle, people take note of that. It does not come right away but it does happen.

### Jennifer’s story

I am 50 years old — born and raised for the most part in Toronto. I was adopted as a newborn but the adoption broke down when I was six years old. Consequently, I grew up in child services, living in many different group homes located throughout Southern Ontario. I started using drugs at a very early age. I can't even remember exactly when. There was a period in my life where I was drug-free during which I went to school, became a nurse and moved to Arizona to practice. While in the States I began using drugs again, lost my license to practice, contracted hepatitis C, went to prison and was eventually deported back to Canada. Upon arriving back in Toronto I had no supports or people I could turn to so I found myself either living in shelters or on the street.

I heard about the Toronto Community Hep C Program from my now husband as he knew someone who had been through it and who had received help applying for and obtaining disability benefits. This interested me since the more money I could get the more I would have for drugs. The TCHCP, of course, wanted to treat my hep C if I was eligible for treatment. Although treatment was not a priority for me, I decided to stay with the program and just kind of ‘went with the flow’ — partially because of the honorarium and the food provided at the weekly group but also because it was the one single thing that I did every week that was good. Everything else I was doing was not so good but this was something positive that I was doing for myself. As it turned out my fibrosis score was high enough (2-3) that I was eligible for treatment. This came as a bit of a shock since I really hadn't had hep C all that long, less than five years, and I wasn't a drinker. I was offered treatment and I thought: “Okay if you want to treat me, what the heck? Let’s do this.” I started treatment for the first time in the spring of 2011 (interferon and ribavarin) but was only a partial responder. The second go-round I was treated with interferon, ribavarin and telaprevir which had to be taken every eight hours and with 30 grams of fat. I just knew that there was no way I could do this particular treatment and still be using drugs. So I quit doing drugs. Thankfully my husband was very supportive and quit with me which made things easier. This time it took — it worked. I am now hep C free. I am blessed. I never felt stigmatized around hep C. Maybe because I didn't have it long enough. I felt stigmatized, more so, for being an injection drug user — specifically. Not just as a drug user, but an injection drug user. That is what I attempted to hide from others and made me feel shame ... not the drugs but the needles.

I was encouraged to join the Patient Advisory Board, the PAB, and did so because I wanted to be more involved and give back to the program that played such a positive role in my life. There was also an honorarium for being on the PAB and every little bit helped in improving my quality of life so I thought, why not? I wanted to apply for the Community Support Worker training but I had lost a lot of confidence in myself. I made the application process into some huge, insurmountable barrier. In the end I applied but missed the deadline and was therefore not given one of the spots. Thankfully a spot opened up a couple of weeks into the training and I got in, completed and graduated. I wasn't sure I wanted the job as a Community Support Worker and was feeling a lot of ambivalence but applied because I felt it was expected of me. Left to my own devices I probably wouldn't have applied but I am so grateful that I was able to get out of my own way and that I did.

My job as a Community Support Worker has changed a lot since I started. I used to help facilitate one of the treatment groups but now I mostly do hepatitis C education for new clients and training for other peer workers. I am driven by wanting to help people access treatment — to get as many people on treatment as possible. It's also a way for me to give back and stay humble. I'm not where I was when I started this journey and I need to remember this and stay grounded. This job, helping others, allows me to do that.

## Results

### Case presentations

Five primary themes emerged during our analysis of the facilitators and challenges in the transition from client to support worker: (1) the role of prior experiences, (2) changes in substance use practices, (3) shifts in relationships with community members and friends, (4) supportive organizational and structural factors, and (5) role transition as a journey. In some cases, key factors contained elements that were both facilitating and challenging.

### Theme 1—the role of prior experiences

Jennifer and Marty brought very different work histories to their role as CSW. Marty had worked primarily in a variety of non-office based labor jobs, and Jennifer had worked as a hospital-based nurse. While Jennifer felt that her past employment experience in a health care setting helped to prepare her for a successful transition to CSW, she also acknowledged that this work history may have also initially been a challenge as she had to unlearn some of the discipline she had been taught as a nurse before she could become comfortable in her new role.

“I realized something the other day … I was a really good nurse because it’s very cut and dry and it has a start and it has an end and this work doesn’t have an end and it doesn’t really have a clear start either, you know what I mean? It’s fluid. Not that nursing cannot be fluid at times … here it’s never ending. It just doesn’t stop … I don’t think I am as good … yeah, I’m not a good social worker [laughs].” Jennifer

Both agreed that Marty’s varied and adaptive work history might have initially been more helpful for the ever-changing and intuitive work of a CSW.

Throughout the interviews and when directly asked to identify what helps someone to successfully make the transition from client to worker, both also identified several personal qualities that they felt were key. Marty identified being non-judgmental, easygoing, and conflict avoidant as qualities he felt were important in his successful transition to peer work, while Jennifer identified her serious predisposition as contributing to her success. Both Marty and Jennifer identified as being “natural helpers” or leaders, partly because of some of these individual qualities but also because of their past experience as founding members of the program’s patient advisory board.

“ … I really realized that, yeah, I know a lot of the people coming into the program and a lot of people were looking up to me at the time because I had helped implement all these different programs [as part of the patient advisory board] and stuff so I figured, well hey, I might as well just keep going with it and see what happens.” Marty

Despite this natural tendency towards and experience with community leadership, both were initially reluctant to apply for the CSW role. Marty was hesitant because he felt it would further complicate his life.

“I didn't even really want the job. I didn't think it would last very long. I thought it would bring more turmoil in my life … Out of the six workers they hired I figured I would have been the first one gone … and I'm still here so I must be doing something right.” Marty

Jennifer had initial ambivalence about taking the job and felt motivated to pursue the position primarily out of feeling like program staff expected her to and had encouraged her to do so and because it was the “next step” in her engagement with the program.

### Theme 2—changes in substance use practices

A change in illicit drug use was identified in both cases as facilitating success in the CSW role. For Jennifer, the transition to becoming a CSW was supported by a transition to abstinence from drugs, which was then reinforcing: “I think being a community support worker has; well it’s probably helped me to stay clean too.” Marty continues to use illicit drugs on a daily basis but notes that he uses differently now and that this has been important to his work as a CSW:

“There’s much better ways of doing … using drugs, so that’s how I practice. Drug, set, setting.”

The lack of change in substance use practices of other clients was cited as a challenge. In both cases, concern was expressed regarding having to witness the potentially unhealthy substance use patterns (such as not having enough money left over for groceries after a period of binge use) of program clients, which produced feelings of frustration and anxiety.

“I just feel like, when are you going to get it? And I want them to get it and I get frustrated that they don't get it and there's nothing I can do about that; you know everybody comes to it in their own time.” Jennifer

### Theme 3—shifts in relationships with community members and friends

Both Jennifer and Marty felt that being able to identify, shift, and maintain new personal or community boundaries was critical to their success as peer workers. In both cases, establishing boundaries also meant making personal changes outside of work. For Marty, this centered mainly on his approach to drug use and conflict resolution:

“I live in the neighbourhood where most of the people are and I still associate with a lot of them. That's been one of the trials and tribulations of this job — is knowing your boundaries, because even when I'm done work, you know, and I'm used to going out partying with these guys. I can't do that as much anymore cause they kind of look up at me as a role model up here or whatever. You want to call it right so I can't be going out there and getting in fights and stuff. I have to be able to walk away from things and even though it's not, I'm not at work I still have to practice that.” Marty

For Jennifer, the job was facilitated by establishing a physical boundary with a move to a new neighborhood. This move, which supported Jennifer’s decision to stop using drugs, was cited as reason for success in the role but was also identified as a challenge in terms of staying connected to the community and feeling “authentic” as a peer.

“I feel like the more clean time I have and the more my life has changed and progressed on from that [client stage of life], I feel like my buy-in is becoming … less.” Jennifer

Jennifer and Marty also cited that identifying and maintaining professional boundaries with clients is an ongoing challenge and area of potential risk. They discussed instances early in their employment where they had crossed a particular boundary without, but with the potential for, negative consequences. Both felt that their ability to critically self-reflect, take ownership of, and learn from these transgressions was key to their long-term success in the role.

### Theme 4—supportive organizational and structural factors

Several organizational and structural factors were also identified throughout both interviews as key to the transition from client to peer worker. At an organizational level, the flexible parameters of the job responsibilities were cited by both Jennifer and Marty as important in their transition, allowing them to each find a niche that felt meaningful because it suited their strengths and interests. Having additional hours to attend team meetings and do administrative or program planning activities was also identified by both as a facilitator.

“… I love to teach, I really like teaching so [the job] changed in that way where they saw that I’m good at it, so I started doing more, like in the groups, like I would teach the Hep C 101.” Jennifer

The support and expectations of other program staff, specifically supervisors/managers and co-workers, was also identified. While the patience and lenience of these staff was noted as a facilitator, Jennifer and Marty also expressed the need for this to be balanced with some firm and high expectations. Both also strongly identified the need for sufficient time in the role as key to their transition.

“Having good bosses [helped with the transition], yeah and plus they were pretty lenient when I first started the job.” Marty

“… I think it was kind of important, like finding that balance between being supportive … and the work expectations. So, you know, I think that’s for me the biggest thing and then just like stick with it because I think it takes time for people to start to feel comfortable in their roles and for people to take it on.” Jennifer

At a structural level, stable housing and the additional income provided by the position were also identified by both as important factors that contributed to their successful transition.

### Theme 5—role transition as a journey

“There’s no road map for it.” Jennifer

“It was all just kind of gradual but everything has changed.” Marty

Throughout both interviews, numerous literal or symbolic journeys were identified. For Marty, attempting to access HCV treatment and arriving at the program itself was a literal journey that took him across Canada. For Jennifer, arriving at the program had also been the result of a literal journey, one of deportation from the United States. The job itself has been a metaphorical journey with its evolving tasks and responsibilities as both attempted to find a niche that felt meaningful within the role’s scope of practice. Both Jennifer and Marty have also undergone substantial transformational life journeys since becoming involved with the program—from unstable to stable living situations, from unstable to healthier relationships, and from unstable to stable patterns of drug use. Both Marty and Jennifer were homeless when they began with the program and each now lives in their own apartment. Jennifer is now married and Marty ended a difficult relationship. However, for both Jennifer and Marty, a more notable transition than that from client to worker was the personal transformation that each attributed to their experience of doing HCV treatment, from not being engaged in the program to being deeply engaged. Both experienced difficult treatment experiences and lengthy program engagement periods as clients during which they each made significant life changes. The client experience was itself a journey and was identified as a period of significant personal transition in each case.

“There was no big change besides getting treated … it was a big build-up to get on treatment and there was a big build-up afterwards to get my health back — energy levels and thinking.” Marty

“There’s been lots of turning points. So, the first would be the fact that I came every week [to group] … getting onto [disability support] was a huge turning point … it allowed me to move, you know, to a better place. Coming to meetings regularly. Yeah that was probably the biggest and then, like, actually doing the Patient Advisory Board. That was probably a bit of a turning point too and then getting married of course was a huge turning point. Yeah, everything like progressively got better.” Jennifer

## Discussion

A goal of this study was to provide a better understanding of the factors that appear to be key in the successful transition from client to peer support worker. The role of past experiences, the ability to shift relationships and manage boundaries, a change in substance use, and organizational and structural supports were themes that were identified. In both cases, the transition from peer to worker was marked by a series of literal or metaphorical journeys. There was considerable overlap and interplay between these themes. For example, the flexibility of the CSW positions (organizational support) allowed each worker to find a niche that suited their skills (past experiences). An understanding of the fluidity and complexity of these areas of facilitation and challenge is critical to improving the selection, training, and support of potential peer workers.

Although both of the peer workers who were interviewed in this study identified being “natural leaders” as helping in their transition to peer work, there was little else in terms of past experiences or personal qualities that they identified in common other than an initial hesitation to pursue the job. Both had very different employment histories, from stable employment in a medical role to a varied job history of only non-office-based jobs. In terms of their substance use, they reflect different ends of the harm reduction continuum with one worker still using drugs and one who no longer does. This finding suggests that a supportive harm reduction philosophy that extends to employment expectations can be applied; that abstinence from drugs does not need to be a requirement for employment, and that substance use can be addressed, if and when it impacts on employment. The overall diversity of Jennifer and Marty’s past experiences and qualifications suggests that having broad eligibility criteria for peer positions is possible and that even seemingly unlikely and reluctant candidates should be encouraged, supported, and given opportunities to succeed.

Examination of these peer workers’ experiences supports other studies which have discussed how peer work is supported or limited by organizational structures and suggests a need for flexible role parameters and/or programming [1, 6, 38, 45–47]. The first year of the CSW role was the most challenging for both participants, and when boundary issues were tested. This was also a period of job evolution when each worker explored a variety of responsibilities and tasks within the CSW scope of practice, which allowed them to learn from challenges, build on their strengths, and develop confidence. It was clear that for the individuals in this study, the transition from client to worker was a gradual process and one that was both made possible by, and helped to support, several other transitions or journeys. Both Jennifer and Marty have made substantial personal life changes since becoming involved with the program. These positive changes both enabled and were further supported by the structure and meaningful activity provided by the peer role. Allowing each person to have enough time to become comfortable and find stability and meaning within the peer role enabled success in these cases. The implications of these case experiences suggest that organizations wishing to employ peer workers should maintain a patient and flexible approach to peer integration with clear but not rigid work expectations. Our findings support other studies which have recommended offering a variety of thresholds for peer involvement to suit individuals at various levels of stability and job readiness [38, 45–47]. These findings also suggest that with adequate training and appropriate support, clients can successfully negotiate any potential risks such as challenging health care or personal boundary situations.

Undergoing treatment and being cured of HCV was noted in both cases as a more significant transformative experience than the transformation from client to co-worker. Both workers described a lengthy role transition period. The lengthy program engagement that the old interferon-based HCV treatment required was likely a major factor impacting the transition from client to worker. In addition, both had been founding members of the program’s patient advisory board and were graduates of its peer training course. By the time both Jennifer and Marty began working as CSWs, they had already established a lengthy and deep commitment to the program and to their own personal well-being and self-development. This suggests that an organizational commitment to community development, capacity building for clients and their involvement in program development might support peer worker integration efforts; a finding that is supported by other studies of peer work models [1, 10, 30, 31, 34, 37, 48]. It is also possible that while this lengthy client relationship with the program was a facilitator, it may also have been a hindrance. A shorter time as clients (or no time at all) might have made the transition quicker since both peer workers and other staff would had been less attached to a previous client-provider dynamic. Although this study did not set out to document the therapeutic impacts of peer work on the workers themselves, a variety of positive outcomes were suggested and should be further studied.

### Limitations

Our findings represent only two individuals’ experiences with the transition from client to worker within a hepatitis C treatment program and may not be generalizable to other peer workers or contexts. This study may be limited by selection bias since only peer workers who were still employed by the program were selected and would be complemented by further research which explores why some individuals are not successful as peer workers; however, this was beyond our current scope. The fact that co-authors were in some cases co-workers might have limited what the author-participants felt comfortable saying. This may be particularly true regarding the co-worker dynamic that was not explored here, and yet is an important component of the peer work experience, which warrants further study. Validity was enhanced by involving the research participants themselves in the analysis and writing. The case study participatory design is meant to provide an in-depth evaluation to identify themes for hypothesis generation or further study. The richness of the case studies in our paper provides information and personal insights that would likely have been lost with other more traditional qualitative study designs.

## Conclusions

This study provides insight into the transition from client to support worker and offers some suggestions for how peer workers may be supported in similar programs. The transition was a gradual and non-linear process and one that was supported by and in turn helped to support a number of other personal transitions. The cases examined here suggest that a model of peer employment with broad qualification criteria, sufficient transition timelines, flexible job responsibilities, a solid investment in the inclusion of people with lived experience, and a harm reduction framework supported the successful integration of current and/or former clients into health care teams.

## Abbreviations

PAB: Patient advisory board
CSW: Community support worker
HCV: Hepatitis C virus
TCHCP: Toronto Community Hep C Program
